# The evolution of Airbnb research: A systematic literature review using structural topic modeling

**DOI:** 10.1016/j.heliyon.2023.e17090

**Published:** 2023-06-08

**Authors:** Kai Ding, Yue Niu, Wei Chong Choo

**Affiliations:** a School of Business Administration, Ningbo University of Finance and Economics, Ningbo, China; b Department of Applied Psychology, University of Nottingham Malaysia Campus, Semenyih, Malaysia; c School of Business and Economics, Universiti Putra Malaysia, Serdang, Malaysia; d Institute for Mathematical Research, Universiti Putra Malaysia, Serdang, Malaysia

**Keywords:** Airbnb, Sharing economy, Peer-to-peer accommodation, Text mining, Topic modeling, Sustainable tourism

## Abstract

This study employs advanced text-mining techniques to offer an in-depth and comprehensive overview of the extensive body of research on Airbnb. By analyzing 1021 articles published in 416 journals spanning the period from 2015 to 2022, this study aims at revealing Airbnb research topics and trends. The results show that the primary focus of academic inquiry regarding Airbnb revolves around two domains: the company's operational practices and its impacts on various domains. Within the realm of Airbnb's operational practices, four distinct research topics emerge as particularly prominent and extensively explored. These encompass the dynamics of ‘trust in Airbnb,’ the formulation and implementation of ‘house rules,’ the mechanisms of governing ‘Airbnb pricing’ strategies, and the critical examination of ‘value creation in Airbnb’ initiatives. Meanwhile, the most researched impacts of Airbnb are on urban tourism, rental housing markets, tourist destinations, and hotels. These spheres have received significant scholarly attention due to the profound implications and transformative effects engendered by Airbnb's disruptive presence in these areas. Moreover, the findings underscore that research pertaining to Airbnb's operational aspects has witnessed a significant increase in popularity over time, indicating a marked shift in the focal points of Airbnb research. Notably, the research topics that have experienced substantial growth include ‘trust in Airbnb,’ ‘Airbnb pricing,’ and ‘impacts on tourist destinations.’ Lastly, this study found that Airbnb-related research articles in hospitality and tourism journals tend to be more delving into industry-specific phenomena and challenges. Conversely, non-hospitality and tourism journals provide a broader coverage of topics related to Airbnb, encapsulating diverse areas of inquiry beyond the boundaries of the industry. This literature review provides valuable insights into existing research on Airbnb and highlights several critical areas for future research.

## 1 Introduction

The sharing economy has been one of the most significant developments in recent years in the field of electronic commerce. It refers to an economic model where individuals share their resources, such as homes, cars, and assets, with others for financial gain [1]. The widespread adoption of mobile devices and ubiquitous internet connectivity has fueled the rise of sharing economy platforms. By leveraging digital technologies, the sharing economy creates new marketplaces that connect users directly with service providers, bypassing traditional intermediaries. This decentralized business model has gained popularity not only for economic reasons but also for its potential to create sustainable and environmentally-friendly communities. By reducing waste, promoting the efficient use of resources, and decreasing the environmental impact of goods and services, the sharing economy model contributes to a better future [2].

One of the most well-known examples of a sharing economy company in the accommodation sector is Airbnb. Established in 2008, Airbnb has revolutionized the industry by providing a platform for individuals to rent out their homes or rooms to travelers. Today, it is one of the largest home-sharing platforms globally, operating in over 220 countries and regions [3]. Airbnb offers property owners the opportunity to list their properties and provides travelers with an extensive selection of unique and affordable accommodations [4]. It has hosted over 800 million guests to date [5]. However, the COVID-19 pandemic had a significant impact on Airbnb's business, leading to a sharp decline in bookings due to travel restrictions and lockdowns. However, as restrictions began to ease in some regions, Airbnb experienced a surge in demand for local and domestic travel. Airbnb's transformative impact on the travel industry, sharing economy, and the global economy is evident in its widespread popularity and the numerous academic studies examining its influence.

Researchers have identified several factors that drive customers to adopt Airbnb services, with low cost being a primary motivator [[6], [7], [8]]. Airbnb offers guests a wide range of rental options, including entire places, private rooms, hotel rooms, and shared rooms, which can provide cost-saving options for solo travelers who opt for shared properties. In addition, group travelers can save money compared to traditional hotels. A NerdWallet analysis revealed that the average Airbnb rental for six people was 33% cheaper than booking three hotel rooms [9]. Additionally, numerous Airbnb listings offer fully equipped kitchens, which can help guests save money on meals. The pursuit of an authentic local experience is another key motivator for many guests to choose Airbnb accommodations. Airbnb's “live like a local” approach offers guests the opportunity to stay in properties that are managed and designed by residents, resulting in unique and authentic experiences [10]. This enables guests to gain an unconventional understanding of the local culture and daily life in their destinations [7]. Social interaction is also a significant aspect of the local experience, as Airbnb guests have the chance to interact with hosts, neighbors, and other guests, creating unforgettable experiences and social benefits [11].

Extensive academic research has focused on the disruptive impact of Airbnb on the hospitality industry and housing market. Several studies have reported mixed findings on the effect of Airbnb on hotel performance, with some indicating a negative impact on hotel revenue in specific cities such as Barcelona [12], Paris [13], and London [14], while others have found no significant impact on hotel revenue [15,16]. Additionally, it has been argued that Airbnb accommodations mostly substitute mid-range hotels, indicating that not all hotel categories are directly affected [17]. Apart from its impact on the hospitality industry, Airbnb has also been found to contribute to the increase in rental prices in cities with high Airbnb density [[18], [19], [20]]. Converting long-term rentals into short-term rentals for tourists reduces the supply of available housing, leading to a rise in rental prices [21]. Moreover, the number of commercial Airbnb hosts entering the business is increasing, creating competition for high-quality properties in the housing market, and driving up rents. The impact of Airbnb on housing markets has raised criticism, leading some cities to impose regulations on short-term rentals to address concerns about safety, zoning, and affordability [21,22]. Despite the criticisms, Airbnb remains a significant player in the sharing economy and is likely to play a vital role in shaping the future of travel and tourism.

While Airbnb has caused disruptions, its significant contributions to the growth of the global tourism industry should not be overlooked. Offering tourist accommodation, it enhances the economic impact of tourism on local communities and residents. Tussyadiah and Pesonen [23] suggested that Airbnb increases the number of destinations chosen, the length of stay, and the number of activities pursued, thereby maximizing tourism spending. In addition, Airbnb plays a crucial role in attracting tourists to a city through the online behavior of hosts and guests. Airbnb hosts often attract potential guests by showcasing images of the neighborhood that guests will visit. Airbnb guests also contribute to creating images of tourist destinations. A statistic shows that between 2008 and the COVID-19 pandemic, Airbnb guests provided over 24,000 descriptions of about 500 urban neighborhoods across cities in the northern hemisphere [24]. Moreover, Airbnb creates entrepreneurial opportunities for property owners, who can use it as a low-risk platform to gain early career experience and generate additional income. Fischer et al. [25] revealed that financial gain and economic independence are important motivators for individuals to become micro-entrepreneurs in the sharing economy.

As research on Airbnb continues to evolve, efforts have been made to review the existing literature [[26], [27], [28]], which provided some understanding of the research status of Airbnb. However, many of these studies relied on conventional qualitative analysis methods to analyze text data. Qualitative analysis of literature is time-consuming, and researchers often compromise the depth and rigor of their reviews by limiting the number of journals and years included [29], or excluding relevant articles from related fields, which can have serious implications for their results and contributions. Moreover, manual coding can be subject to subjective bias, as researchers decide what to include and exclude in a topic, which can lead to challenges in interpretive bias and coding objectivity [30]. Traditional methods also limit large-scale reviews of Airbnb literature. Additionally, most previous studies only sample journals from the hospitality and tourism fields, potentially overlooking important insights from non-hospitality and tourism journals.

This study aims to bridge the gap in the literature by conducting a systematic review of articles on Airbnb published in both hospitality and tourism and non-hospitality and tourism journals using text-mining techniques. Text mining has been widely recognized for its ability to synthesize information and identify emerging trends in research topics [[31], [32], [33]]. Unlike manual analysis, text mining provides a scientific basis and eliminates the subjectivity that can arise from group experts [34]. Additionally, text mining allows for the efficient processing of large amounts of text documents, which enables researchers to analyze a larger sample size of literature. This study specifically utilized the Structural Topic Model (STM) tool developed by Roberts et al. [35] to identify key research topics related to Airbnb. This study attempted to address the following research questions:

RQ1: What are the prominent topics that are discussed in research concerning Airbnb?
RQ2: How do the research topic distributions vary across hospitality and tourism journals and non-hospitality and tourism journals?
RQ3: How does the popularity of identified research topics change over time?

This study contributes to the Airbnb research domain by offering a comprehensive understanding of the prominent research topics and trends in this area. This contribution enables academic researchers to identify recent research directions and thereby advance the state of knowledge in this field. This study utilizes both traditional research synthesis and integration methods, as well as a novel approach based on the STM technique, to extract and visualize potential research topics and their evolution over time. The innovative STM approach discussed in this study delves deeper into the research literature and extracts research topics in an automated data-driven manner.

The present study is organized as follows: Section 2 describes the search criteria used to identify relevant research articles on Airbnb, including the inclusion and exclusion criteria, and provides a detailed explanation of the research procedures employed. In Section 3, an overview of the identified articles is presented in terms of their publication journals, year of publication, and thematic coverage, along with an exploration of the interrelationships between various concepts. Section 4 provides a thorough discussion of the findings and implications. Section 5 concludes this study, offering a critical evaluation of the limitations encountered and providing valuable recommendations for future research directions.

## 2 Research methodology

This article adopts a systematic literature review methodology, as indicated by Gupta et al. [36], to analyze literature on Airbnb published in both hospitality and tourism journals, as well as non-hospitality and tourism journals. The Scopus database was selected as the search tool due to its broader coverage of related literature compared to the commonly used Web of Science. The authors used search keywords such as “Airbnb,” “peer-to-peer (P2P) accommodation,” and “sharing economy” in the title, abstract, or keywords to retrieve relevant articles. After manual filtering of articles without abstracts and those not related to the accommodation industry, the authors collected 1021 articles published between October 2015 to October 2022. The Scopus database was the primary data source for this research, and a comprehensive Excel file containing research titles, journal titles, abstracts, keywords, publication dates, and author affiliations was downloaded and stored for analysis.

**Fig. 1:**
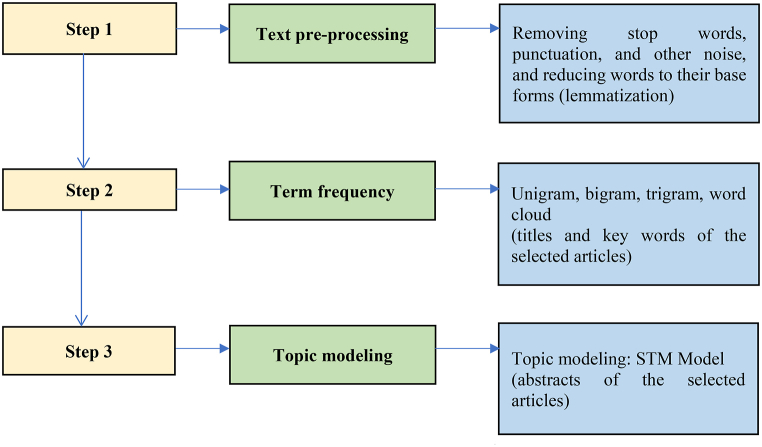
Research procedures. Source: Created by the authors.

Fig. 1 outlines the research procedures employed after data collection. The first step is text pre-processing, which involves converting all text to lowercase and removing special characters such as punctuation, numbers, and white space that are irrelevant to the text-mining analysis. A set of commonly used English words, also known as stop words, are eliminated from the document using the SMART lexicon in the “TM” package to focus on words that are more meaningful to researchers. In addition, a customized list of stop words, such as publisher names and abstract sub-titles, is utilized to remove any remaining stop words. The words are then lemmatized to normalize different word forms to a common base form, reducing the corpus's dimensionality. The advantage of lemmatization over stemming is that it considers the context of a word to determine its intended meaning, avoiding over-stemming that could result in the loss of important information and undesirable interpretability [37]. The abstracts serve as input for topic modeling, and frequent bigrams in the abstracts are concatenated. In Step 2, the term frequency method is used to analyze the article titles and keywords. The frequency distribution of unigrams, bigrams, and trigrams is computed, and a word cloud technique is utilized to show frequently occurring words in the documents. The larger the font in the word cloud, the more frequently the word appears in the document, and vice versa.

In Step 3, this study utilizes STM topic modeling to identify major research topics on Airbnb by analyzing abstracts of selected articles. STM is a unique type of topic modeling specifically designed to meet the research needs of social scientists. It provides the ability to integrate metadata into the model and to discern how different documents express a shared underlying topic using varying lexical constructs [38]. As a valuable tool, STM provides several advantages for literature reviews. It enables the identification and exploration of latent topics in a given corpus, facilitating the discovery of themes and patterns that may not be immediately apparent through traditional methods like qualitative reviews. Additionally, STM allows for the incorporation of document-level metadata as covariates [35], which helps examine the relationship between topics and external factors such as publication year, author, and document type. In this study, the covariate is the field of the journal, i.e., hospitality and tourism, and non-hospitality and tourism. STM's framework is highly flexible, making it possible to analyze large and complex datasets, including handling missing data. Finally, STM enables the estimation of topic prevalence over time, making it possible to explore how research topics on Airbnb evolve and change over time.

**Fig. 2:**
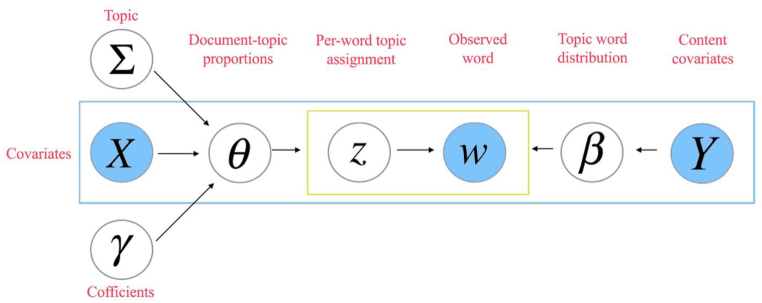
Illustration of the structural topic model. Source: Adopted from Roberts [38].

Fig. 2 depicts the generative model, which starts by considering a document, represented by *d* ∈ 1 … *D*, and the words contained within the document, denoted by *n* ∈ 1 … *N*_*d*_. These words, represented as *w*_*d*,*n*,_ are treated as unique terms from a vocabulary of size *V*, indexed by *v* ∈ 1 … *V*. Additionally, the model incorporates *K* topics, where k ∈ 1 … *K*. Two design matrices, *X* and *Y*, provide further observed information regarding the topic prevalence and topical content, respectively. Researchers can specify these matrices based on a document and its associated covariate vector for each row. The dimension of *X* is *D* × *P*, whereas *Y* has a dimension of *D* × *A*, and their respective rows are denoted as *x*_*d*_ and *y*_*d*_. Finally, the model considers the marginal log frequency of term *v* in the vocabulary, which is estimated from total counts, and denoted as *m*_*v*_.

The generative process of a document in a STM model with *k* topics can be described using four scalar hyper-parameters (*s*, *r*, *ρ*) and a *K*-dimensional hyper-parameter vector (*σ*) as:

$$\begin{array}{c} \gamma_{k}∼{Normal}_{P}\left(0,\sigma_{k}^{2}I_{P}\right),for\;k=1\ldots K-1\text{,}\\ \theta_{d}∼{LogisticNormal}_{K-1}\left(\Gamma^{\prime }x_{d}^{\prime },\Sigma \right)\text{,}\\ z_{d,n}∼{Multinomial}_{K}\left(\theta_{d}\right),for\;n=1\ldots N_{d}\text{,}\\ w_{d,n}∼{Multinomial}_{V}\left(B_{d,n}\right),for\;n=1\ldots N_{d}\text{,}\\ \beta_{d,k,v}=\frac{\exp\left(m_{v}+k_{k,v}^{\left(t\right)}+k_{y_{d},v}^{\left(c\right)}+k_{y_{d},k,v}^{\left(i\right)}\right)}{\Sigma_{v}\;\exp\left(m_{v}+k_{k,v}^{\left(t\right)}+k_{y_{d},v}^{\left(c\right)}+k_{y_{d},k,v}^{\left(i\right)}\right)},for\;v=1\ldots Vandk=1\ldots K\text{,} \end{array}$$

where the logistic normal distribution is utilized by the core language model to establish associations among topic proportions. For a model comprising *K* topics, the logistic normal distribution is denoted by *η*_*d*_ ∼ Normal_*K*−1_ (*μ*_*d*_, Σ) and mapped onto the simplex with $\theta_{d,k}=\exp\left(\eta_{d,k}\right)/\left(\Sigma_{i=1}^{K}\;\exp\left(\eta_{d,i}\right)\right)$. In order to ensure model traceability, *η*_*d*_*,*_*k*_ is set to zero. For every word in document *d*, a topic is randomly selected from the multinomial distribution *z*_*d,n*_ ∼ multinomial (*θ*_*d*_), based on the given topic proportion vector, *θ*_*d*_. The word is chosen from the distribution of terms B_*zd,n*_, conditioned on the topic and written as *β*_*zd*,*n*_ for simplicity.

## 3 Findings

### 3.1 Overview of selected journals

Table 1 displays a ranking of the top journals that published Airbnb-related studies, with the number of appearances and percentage of total appearances listed. The International Journal of Hospitality Management appears the most, with 75 appearances, representing 18% of the total. The International Journal of Contemporary Hospitality Management, Sustainability (Switzerland), and Tourism Management all have 36 appearances, representing 8.8% of the total. The Annals of Tourism Research has 27 appearances, representing 6.4% of the total, while the Journal of Hospitality and Tourism Management has 18 appearances, representing 4.3%. The remaining 11 journals have between 11 and 14 appearances, representing between 2.6% and 3.3% each, and the “Others” category represents 48 appearances or 11.5% of the total. Among the journals that published more than 10 articles related to Airbnb, Sustainability, Environment and Planning A and Journal of Business Research are not under the category of hospitality and tourism. Notably, several journals that have published over 10 articles on Airbnb, such as Sustainability, Environment and Planning A, and Journal of Business Research, are not classified as hospitality and tourism journals. This suggests that research on Airbnb extends beyond the hospitality and tourism industry and has broader implications for sustainability and business research.

**Table 1:** Number of selected articles by journal.

No.	Source title	Frequency	Percent (%)
1	International Journal of Hospitality Management	75	18.0
2	International Journal of Contemporary Hospitality Management	37	8.8
3	Sustainability (Switzerland)	36	8.6
4	Tourism Management	36	8.6
5	Annals of Tourism Research	27	6.4
6	Journal of Hospitality and Tourism Management	18	4.3
7	International Journal of Culture, Tourism, and Hospitality Research	17	4.0
8	Tourism Economics	17	4.0
9	Journal of Sustainable Tourism	15	3.6
10	Journal of Travel Research	15	3.6
11	Current Issues in Tourism	14	3.3
12	International Journal of Tourism Cities	14	3.3
13	Journal of Travel and Tourism Marketing	14	3.3
14	Environment and Planning A	11	2.6
15	Journal of Business Research	11	2.6
16	Tourism Management Perspectives	11	2.6
17	Others	48	11.5
	Total	416	

Note: The Journals shown in the table refer to the samples collected in this study from the Scopus database.

### 3.2 The publication trend of Airbnb research

**Fig. 3:**
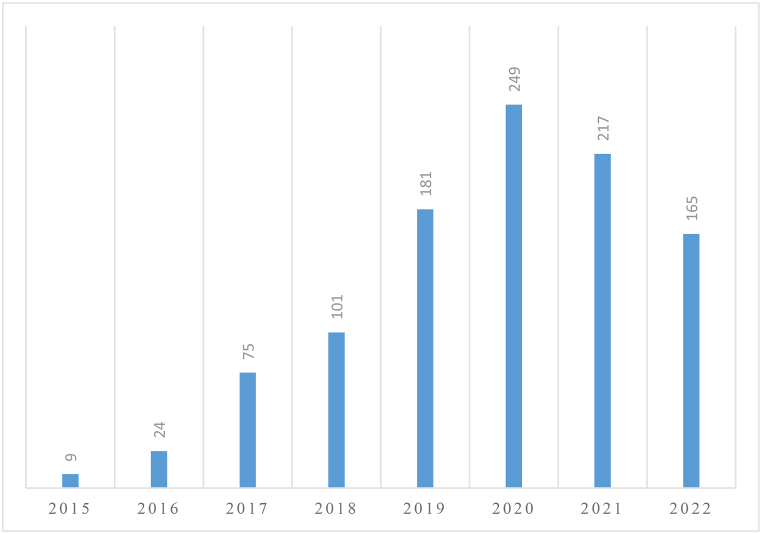
Article count trend. Source: Created by the authors.

Based on Fig. 3, there has been a remarkable increase in the annual quantity of academic articles focusing on Airbnb over the years. This growth has been particularly significant since 2016, with a peak of 249 publications in 2020, followed by a slight decline in the following two years. The rise in academic interest in Airbnb can be attributed to the rapid growth and expansion of the sharing economy, in which Airbnb plays a prominent role. Scholars from diverse fields, including business, economics, and hospitality, have been increasingly interested in exploring the impacts of the sharing economy on various aspects of society. The decline in Airbnb-related publications after 2020 may be attributed to the pandemic's effect on the travel and tourism sector. Due to the widespread disruption of travel and tourism activities, including border closures, lockdowns, and restrictions on public gatherings, research on Airbnb and its implications may have been deprioritized or delayed. Nevertheless, given the enduring significance of the sharing economy and its potential for growth and disruption, researchers are likely to continue investigating Airbnb's impact on various domains of society in the future.

### 3.3 N-gram analysis

N-gram analysis is a statistical method used to detect repeated word patterns in a body of text [39]. Table 2 displays the result of an N-gram analysis of Airbnb research keywords. The most frequent unigram is “tourism,” indicating that Airbnb research places significant emphasis on the tourism industry. This is followed by “analysis,” “industry,” “market,” and “tourist,” which suggest that Airbnb is interested in examining the tourism industry and market. The prevalence of terms such as “social,” “behavior,” and “urban” suggests that Airbnb research also focuses on the social and behavioral aspects of tourism, particularly in urban areas. The most frequent bigrams and trigrams provide further insight into Airbnb's research focus. For instance, “tourist_destination” and “tourism_economics” indicate Airbnb's interest in the economic consequences of tourism and how tourists select their destinations. “Hospitality_industry” and “hotel_industry” suggest that Airbnb is also interested in the competition between traditional hotels and the sharing economy. Furthermore, the presence of terms such as “world_wide_web,” “distributed_computer_systems,” and “human_computer_interaction” suggests that Airbnb research also investigates the technological aspects of the tourism industry. The frequent use of location-specific terms such as “new_south_wales,” “ile_de_france,” and “ville_de_paris” indicates that Airbnb research explores the regional variations of tourism.

**Table 2:** N-grams analysis of research keywords.

No.	Unigram	Fre.	Bigram	Fre.	Trigram	Fre.
1	tourism	233	tourist_destination	65	world_wide_web	15
2	analysis	162	united_states	64	new_south_wales	9
3	industry	116	tourism_economics	57	ile_de_france	8
4	market	104	hospitality_industry	54	ville_de_paris	8
5	tourist	104	hotel_industry	51	distributed_computer_systems	5
6	social	102	rental_sector	50	santa_cruz_de	4
7	behavior	94	tourism_market	46	cruz_de_tenerife	4
8	urban	90	sharing_economy	39	human_computer_interaction	3
9	economics	89	tourism_development	38	new_york_city	3
10	development	82	tourism_management	32	decision_support_systems	3
11	economic	80	new_york	30	deep_neural_networks	3
12	sector	79	tourist_behavior	28	convolutional_neural_network	3
13	united	76	social_media	28	major_clinical_study	3
14	data	74	housing_market	25	natural_language_processing	2
15	economy	73	spatial_analysis	25	artificial_neural_network	2
16	housing	73	service_sector	24	least_squares_method	2
17	internet	72	urban_area	23	sustainable_development_goal	2
18	destination	66	consumption_behavior	21	randomized_controlled_trial	2
19	states	64	decision_making	20	data_envelopment_analysis	2
20	information	58	price_dynamics	19	channel_state_information	2
21	research	57	service_quality	16	electronic_document_exchange	2
22	sharing	56	data_mining	16	mean_square_error	2
23	service	56	empirical_analysis	16	time_series_analysis	2
24	hospitality	55	travel_behavior	16	social_media_analytics	2
25	management	54	world_wide	15	propensity_score_matching	2
26	rental	53	wide_web	15	cutting_edge_technology	2
27	hotel	51	urban_housing	15	social_sciences_computing	2
28	new	50	social_network	15	consumer_health_information	2

Note: The keywords listed in this table are the specific terms and phrases extracted from the articles selected from the Scopus database for this study.

Table 3 displays the frequency of n-grams in a corpus of Airbnb research titles. The most frequent unigram is “Airbnb” (502 occurrences), which suggests that the majority of research focuses on Airbnb as a platform. The second most frequent unigram is “sharing” (226 occurrences), highlighting the importance of the sharing economy concept in research. The most frequent bigram is “sharing_economy” (172 occurrences), emphasizing the significance of this concept. The second most frequent bigram is “Airbnb_listings” (22 occurrences), indicating that research has also explored the characteristics of Airbnb listings. Regarding trigrams, the most frequent is “New_York_City” (nine occurrences), suggesting that New York City is a popular location for Airbnb research. The second most frequent trigram is “sharing_economy_platform” (five occurrences), indicating an interest in studying different platforms that are part of the sharing economy. Other frequent terms in the corpus include “economy,” “accommodation,” “tourism,” “platform,” and “case,” demonstrating the different aspects of Airbnb research that have been explored. The results suggest that the sharing economy and Airbnb as a platform are major areas of interest in the research community. Moreover, the frequent occurrence of terms related to accommodation and tourism suggests that research has investigated the impact of Airbnb on these industries.

**Table 3:** N-grams analysis of research titles.

No.	Unigram	Fre.	Bigram	Fre.	Trigram	Fre.
1	airbnb	502	sharing_economy	172	new_york_city	9
2	sharing	226	airbnb_listings	22	sharing_economy_platform	5
3	economy	213	case_study	18	geographically_weighted_regression	4
4	accommodation	116	airbnb_hosts	18	sharing_economy_platforms	4
5	tourism	72	collaborative_consumption	13	service_quality_attributes	3
6	platform	70	airbnb_guests	12	using_airbnb_data	3
7	case	69	sharing_accommodation	10	de_airbnb_en	3
8	analysis	66	platform_economy	10	business_model_innovation	3
9	platforms	59	new_york	10	airbnb_listing_prices	3
10	online	57	online_reviews	10	systematic_literature_review	3
11	impact	55	tourist_accommodation	10	text_mining_approach	2
12	evidence	54	hotel_industry	10	hedonic_pricing_model	2
13	study	51	european_cities	10	consumer_loyalty_toward	2
14	trust	46	service_quality	9	loyalty_toward_airbnb	2
15	hosts	45	repurchase_intention	9	revenue_management_practices	2
16	social	45	york_city	9	airbnb_online_reviews	2
17	cities	44	housing_market	9	hedonic_pricing_approach	2
18	rental	41	p2p_accommodation	8	fourth_industrial_revolution	2
19	effects	39	price_determinants	8	online_collaborative_consumption	2
20	new	39	social_media	8	new_car_sales	2
21	service	38	airbnb_users	8	social_exchange_theory	2
22	urban	38	hotel_performance	7	exchange_theory_perspective	2
23	effect	38	accommodation_platforms	7	airbnb_spatial_distribution	2
24	digital	37	tourism_industry	7	collaborative_consumption_platforms	2
25	market	36	spatial_distribution	7	technology_acceptance_model	2
26	exploring	36	business_model	7	casa_alle_zattere	2
27	hotel	34	airbnb_host	7	book_accommodation_via	2
28	housing	34	collaborative_economy	7	accommodation_via_online	2
29	host	34	empirical_evidence	7	barcelona_e_lisboa	2
30	reviews	34	airbnb_reviews	6	um_estudo_comparativo	2
31	spatial	34	empirical_study	6	automatically_debugging_memory	2
32	city	34	airbnb_accommodation	6	debugging_memory_leaks	2
33	approach	32	economy_platform	6	platform_business_model	2
34	using	32	rental_platforms	6	urban_tourism_context	2

Note: The terms and phrases presented in this table are taken from the article titles that were selected from the Scopus database for this study.

### 3.4 Topic number determination

In the context of STM topic modeling, the selection of an appropriate number of topics, denoted by *K*, is critical for ensuring the reliability and accuracy of the results obtained. Previous studies have suggested that both quantitative and qualitative criteria should be employed in the selection of the optimal number of topics [38]. The quantitative criterion for determining the optimal number of topics is based on a trade-off between two statistical metrics: topic coherence and exclusivity. Topic coherence is a measure of the semantic relatedness between words that tend to appear in the same topic, and it is estimated by computing the sum of the log ratio between co-document frequency and the document frequency for the most likely words within a topic [40]. This metric assumes that words that are semantically related are likely to co-occur frequently within the same document. Exclusivity, on the other hand, is a measure of the distinctiveness of different topics, and it is computed by comparing the similarity of the word distributions of different topics [35]. High exclusivity indicates that more words belong exclusively to a specific topic. Despite the benefits of using quantitative criteria to determine the optimal number of topics, it is not always consistent with the choices made by human coders [41]. Therefore, researchers are encouraged to conduct a qualitative examination of the relevance and interpretability of the identified topics within their specific research context.

**Fig. 4:**
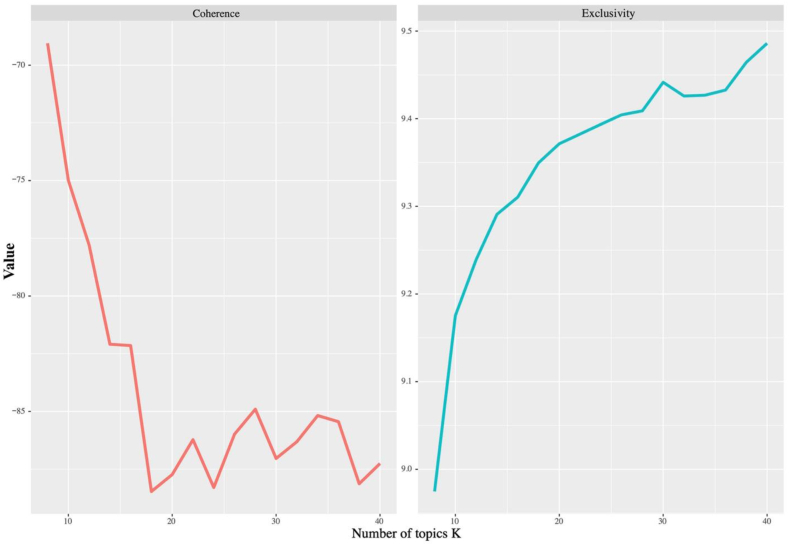
The semantic coherence and exclusivity of 40 topic models. Note: The semantic coherence score measures the degree of semantic similarity among the topics generated by the model. The exclusivity score quantifies the exclusiveness of topics, highlighting the extent to which a particular topic is distinct from others. Source: Created by the authors.

We examined the topic coherence and exclusivity of different topic model solutions within the range of 10–40 topics. Fig. 4 illustrates the results; the topic coherence on the left and exclusivity on the right, with the smaller semantic coherence values indicating better topic model performance, and larger exclusivity values indicating better topic model performance. However, none of the topic models demonstrated superior performance on both statistical measures. We also examined the interpretability of the models for topic models ranging from 18 to 28 to determine the most suitable number of topics. After thorough analysis, we determined that the optimal number of topics was 20. This decision was based on the fact that beyond 20 topics, there was no significant improvement in both topic coherence and exclusivity. Moreover, the model with 20 topics had more interpretable topics and fewer overlapping topics.

### 3.5 Topic labeling and evaluation

**Fig. 5:**
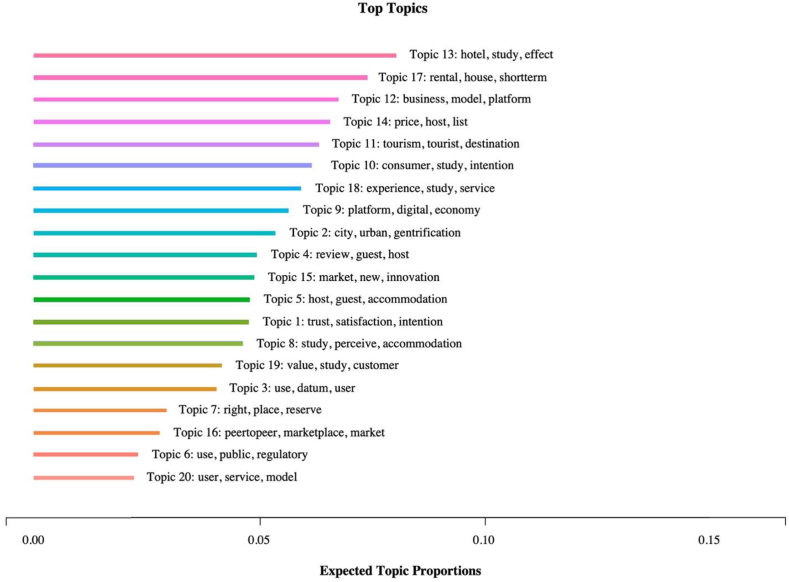
Ranking of topics by proportions. Note: The ranking presented in this figure is determined by the expected proportions of the identified topics, which indicates the relative significance and prevalence of different topics within the analyzed dataset. Source: Created by the authors.

The *LabelTopics* function, a part of the “STM” package in R language, enables the labeling of identified topics by creating a comprehensive list of topic-related terms with varying degrees of importance, as defined by four distinct weightings: Highest Probability, FREX, Lift, and Score. Of these weightings, the Highest Probability and FREX rankings were primarily utilized to identify the most appropriate labels for each topic, as they reflect the most frequent and distinctive terms associated with a given topic. To ensure the accuracy and reliability of the labeling process, the final selection of each label is subjected to a rigorous verification process. This involves a comprehensive evaluation of each candidate label based on its relevance, specificity, and accuracy in capturing the main themes and patterns within the topic. To better understand the context of the top words that appear in each topic, the *findThoughts* function was used to generate a representative sample of text. This sample can help researchers identify the main themes and patterns within each topic and create a list of potential labels. The final decision on the most appropriate label in this study is made through a collaborative process among the researchers, where consensus is reached through discussion and deliberation. Fig. 5 illustrates a ranking of the extracted topics based on their proportions. The proportions refer to the percentage of total occurrences of each topic in the dataset. Each topic is represented by a number, and the figure also displays the three most frequently occurring words within that topic. The quality of each topic is assessed by plotting semantic coherence and exclusivity scores, illustrated in Fig. S1 (Supplementary material). Among the 20 topics, ‘technological innovation’ scored lowest for exclusivity, and ‘Airbnb cybersecurity’ scored lowest for semantic coherence. Hence, more attention should be paid when interpreting these two topics. The resulting labels for each topic and the top words ranked by the Highest Probability and FREX metrics are presented in Table 4.

**Table 4:** Topic summary.

No.	Topic Label	Proportion	Top 7 words	
			Highest Probability	FREX
1	Trust in Airbnb	4.73%	trust, satisfaction, intention, platform, study, customer, service	trust, satisfaction, repurchase, antecedent, recovery, loyalty, intention
2	Impacts on urban tourism	5.32%	city, urban, tourism, gentrification, tourism, analysis, datum	gentrification, urban, neighborhood, historic, barcelona, touristification, concentration
3	Data applications	3.99%	use, datum, user, model, information, system, propose	prediction, accuracy, label, dataset, machine, crowdsourcing, neural
4	Airbnb website content analysis	4.92%	review, guest, host, information, platform, rating, online	rating, photo, review, description, score, decision, sentiment
5	Host-guest interactions	4.75%	host, guest, accommodation, hospitality, tourism, study, network	hospitality, host, traveler, interview, couchsurfing, network, upon, book
6	House rules	2.27%	use, public, regulatory, venue, host, study, smoke	smoke, venue, permit, prevalence, regulate, fire, tax
7	Airbnb cybersecurity	2.87%	right, place, reserve, paper, leak, use, make	leak, residence, web, bleak, right, law, memory
8	Motivations of Airbnb users	4.61%	perceive, accommodation, intention, impact, use, tourist, resident	traveler, perceive, attitude, behavioral, perception, intention, towards
9	Governance of sharing economy platforms	5.62%	platform, digital, economy, market, article, social, control	digital, governance, labour, driver, democratic, algorithmic, infrastructure
10	Perceived risk	6.24%	consumer, study, intention, effect, user, risk, perceive	consumer, brand, risk, behavioral, motivation, gender, identity
11	Impacts on tourist destinations	6.28%	tourism, tourist, destination, study, accommodation, impact, development	destination, tourism, cluster, conflict, overtourism, coastal, tourist
12	Business model analysis	6.71%	business, model, platform, share, innovation, disruptive, economy	business, multisided, innovation, disruptive, firm, sustainability, company
13	Comparisons of Airbnb and hotels	8.01%	hotel, study, effect, demand, market, price, impact	hotel, demand, occupancy, spatial, distribution, location, rate
14	Airbnb pricing	6.53%	price, host, listing, attribute, performance, use, effect	listing, price, attribute, signal, performance, revenue, listing
15	Technological innovation	4.85%	market, new, innovation, technology, uber, create, design	innovation, transport, institutional, student, technology, society, uber
16	Buying behavior	2.75%	peertopeer, marketplace, market, individual, peer, personal, seller	seller, buyer, peer, marketplace, personal, selfpresentation, product
17	Impacts on housing prices and rents	7.31%	rental, house, shortterm, market, city, increase, impact	rental, shortterm, house, rent, longterm, residential, regulation
18	Airbnb user experience analysis	5.91%	experience, service, review, host, customer, accommodation, guest	experience, topic, authenticity, dimension, theme, extract, quality
19	Value creation in Airbnb	4.12%	value, customer, consumption, social, experience, online, cocreation	cocreation, value, consumption, collaborative, customer, dimension, proposition
20	Service preferences of users	2.16%	user, service, model, method, analysis, propose, preference	preference, procedure, match, crowd, nature, method, user

Note: The highest probability words are the terms that are most likely to be connected with a particular topic. FREX is a measurement that considers how often words appear in a specific topic and how unique they are to that topic compared to others.

### 3.6 Topic interpretation

In this section, we aim to provide an explanation of each topic by referring to the top words and prior studies. Topic 1 focuses on the trust mechanism of Airbnb, which is closely related to user satisfaction and loyalty behavior. The keywords associated with this topic include “trust,” “satisfaction,” and “repurchase.” Previous researchers investigated the relationship between trust and user satisfaction and loyalty behavior. Chuah et al. [42] found that perceived trust has a positive impact on user satisfaction with Airbnb, which in turn, increases their intention to repurchase. Similarly, Lee and Kim [11] reported that trust has a significant positive effect on user satisfaction and behavioral loyalty towards Airbnb. Topic 2 is related to the impact of Airbnb on urban tourism, with the most frequent words being “city,” “urban,” and “tourism,” while the related FREX words include “touristification” and “overtourism.” Previous studies have explored the effects of Airbnb on urban tourism, indicating that the platform has both positive and negative impacts on cities. On the positive side, Airbnb has been shown to increase tourism and stimulate local economic development. However, it has also been associated with issues such as over-tourism and changes in the character of urban neighborhoods. In particular, the phenomenon of “touristification” has been identified as a concern in some cities, where Airbnb and other short-term rental platforms have led to an increase in tourist-oriented businesses and a decline in local culture [15,43].

Topic 3 is concerned with the application of Big Data techniques in the context of Airbnb for different purposes, such as algorithm development, prediction, and knowledge discovery [44,45]. Big Data has been recognized as a valuable resource for businesses, including those in the hospitality industry, as it provides access to large amounts of data that can be analyzed to identify patterns, trends, and insights. Topic 4 is about the analysis of the Airbnb website content for various purposes, ranging from understanding the platform's user behavior to developing strategies for successful listing management. The investigation into the Airbnb website's content involves a comprehensive analysis of various data types, including textual data generated by customers' reviews, ratings, profile photos, and geolocation data.

Topic 5 explores the role of host-guest interactions in shaping the guest experience and the overall success of hosting. These interactions occur before, during, and after the stay and have been linked to higher guest satisfaction, ratings, and bookings [15,23]. Kim et al. [46] highlighted the importance of hosts being responsive and communicative during the pre-stay stage to receive positive reviews and repeat bookings. However, effective host-guest interactions are not always straightforward. They can be complicated by cultural differences, language barriers, and varying expectations, leading to misunderstandings [47]. Topic 6 focuses on setting rules for using Airbnb's lodging services, particularly those related to user safety. Safety concerns have been found to be a significant factor in guests' decision-making process when selecting an Airbnb property and have been linked to guest satisfaction with their Airbnb experience [22,48].

Topic 7 focuses on research related to Airbnb cybersecurity, specifically on detecting and analyzing web leaks on the Airbnb platform. This is a niche but essential aspect of Airbnb research, as it aims to identify potential vulnerabilities in the website and analyze past incidents of leaks, breaches, or other security incidents to ensure the safety and privacy of users. Topic 8 is focused on investigating the different factors that motivate users to adopt the Airbnb service. Research has shown that there are several key factors that influence users’ decision to use Airbnb over traditional hotels. So et al. [49] found that price value, enjoyment, and home benefits were important factors that led to the adoption of Airbnb. Other factors that have been found to motivate users to adopt Airbnb include social influence, convenience, and the desire for unique and authentic experiences [15]. For example, users may be motivated to use Airbnb because their friends or family members have recommended it, or because it offers a more convenient or flexible travel option than traditional hotels. Additionally, users may be attracted to Airbnb because it allows them to stay in unique and distinctive properties that offer a more authentic and immersive travel experience.

Topic 9 focuses on the governance of various sectors within the sharing economy, including transportation and hospitality, which have disrupted conventional business models. It is suggested that the sharing economy requires a new regulatory paradigm that balances the need to safeguard consumers with the need to promote innovation and competition [50]. Topic 10 concerns the perceived risks associated with using Airbnb as a consumer and how this impacts their willingness to use the platform. Several studies have explored users' perceptions of the risks linked with Airbnb, such as safety concerns [51], host reliability [52], and the experience of staying in a stranger's home [53]. Another stream of studies explored the impact of perceived risks on consumer behavior and their motivation to choose Airbnb over traditional hotel options [54,55].

Topic 11 delves into the impact of Airbnb on tourist destinations from various perspectives, such as economic growth, environmental sustainability, and tourism destination management. Frequently used keywords that can help to define this topic include “tourism,” “tourist,” “destination,” and “impact.” The impact of Airbnb on tourist destinations is a multifaceted issue that has been extensively researched. While the growth of Airbnb and other home-sharing platforms can provide new opportunities for local entrepreneurs and generate additional income for residents, concerns have been raised about the rise of short-term rentals leading to increased housing costs and reducing the availability of long-term housing, which can negatively impact the local economy [21]. Additionally, the environmental impact of short-term rentals, especially in areas with fragile ecosystems, has become a growing concern. Topic 12 focuses on analyzing the P2P business model, which is a key characteristic of numerous sharing economy platforms. To illustrate and contrast the diverse P2P companies, Airbnb is frequently used as a case study.

Topic 13 focuses on the comparison between Airbnb and traditional hotels. Given the similarities in the nature of service, researchers have compared Airbnb with traditional hotels from various perspectives, such as accommodation pricing [7], spatial distribution [56], contribution to tourism [16], and revenue growth [57]. Topic 14 focuses on studies related to the pricing of Airbnb listings. These studies examine the various factors that influence the price of Airbnb listings and explore pricing strategies that hosts can use. For example, location, property type, availability, and the host's experience level are identified as factors that significantly impact the price of Airbnb listings [58]. In addition, factors such as listing type, room size, location, and seasonality also affect the price [59,60]. Researchers have also explored various pricing strategies that hosts can use to maximize their earnings on Airbnb. For instance, one study proposed a pricing model that considers profile characteristics [61], while another suggested a dynamic pricing approach that enables hosts to adjust their prices in response to changes in supply and demand [62].

Topic 15 focuses on the use of advanced digital technologies and how they can improve organizational performance and address challenges, using Airbnb as a case study. Bolton et al. [63] highlighted the integration of emerging technologies like artificial intelligence and blockchain into Airbnb's platform to enhance the guest experience and host management. Topic 16 investigates the factors that influence the online purchasing behavior of users on the Airbnb platform, with a focus on both host and property attributes. Host attributes include factors such as reputation, credibility, responsiveness, and profile completeness [64,65], while property attributes refer to descriptions and the property's ability to meet guests' specific needs.

Topic 17 explores the impact of Airbnb on the housing and rental markets. Studies have shown that a 10% increase in Airbnb listings in a city leads to a 0.39% increase in rents and a 0.64% increase in house prices [66], as well as having a significant and positive impact on local housing prices [19]. The legal and regulatory perspective of Airbnb's impact on the housing market has also been discussed. Huang et al. [67] highlighted the need for effective regulation of short-term rentals to avoid the negative effects they can have on the housing market, while Nieuwland and Van-Melik [68] argue that local governments should implement policies that balance the economic benefits of short-term rentals with the potential negative impacts on the availability and affordability of housing for local residents. Regarding the impact of Airbnb on rents, Gurran and Phibbs [21] found that the proliferation of Airbnb has caused a significant surge in short-term rentals, which may limit the availability of long-term rental housing and increase rental costs. Liang et al. [69] discovered that the expansion of Airbnb has resulted in the displacement of long-term renters and a reduction in the availability of cost-effective rental housing, resulting in negative social and economic consequences in neighborhoods. Topic 18 covers a wide range of user experience aspects, including customer service, amenities, and cleanliness. An interesting trend in recent user experience research on Airbnb is the increasing use of online review data mining as a methodology.

Topic 19 examines the concept of value creation in the context of Airbnb, with the word “value” appearing in both the Highest Probability and FRX word lists. Most studies exploring Airbnb's value creation are from the perspective of collaborative consumption in the sharing economy, with different values being discovered based on users' subjective perceptions. Tussyadiah and Zach [70] suggested that Airbnb hosts create value for guests by providing unique and personalized experiences, while guests create value for hosts by providing social interaction and supplemental income. Additionally, researchers have identified various types of value that Airbnb provides, including economic, social, and environmental value [7,71]. Topic 20 focuses on the preferred services of Airbnb users. Preferred services refer to the features and amenities that hosts offer on the platform, which are most desirable to guests. Understanding these preferences can help hosts tailor their offerings and improve the overall guest experience [72]. It is important to note that preferences may vary depending on the location, type of property, and guest demographics [16,73]. In addition, previous studies have also discussed service recovery. Tussyadiah and Pesonen [23] emphasized that service recovery is critical in determining user satisfaction with Airbnb. Users who have experienced a service failure but received a prompt and effective response from the company are more likely to have a positive view of the platform and recommend it to others.

### 3.7 Topic distribution comparison analysis

**Fig. 6:**
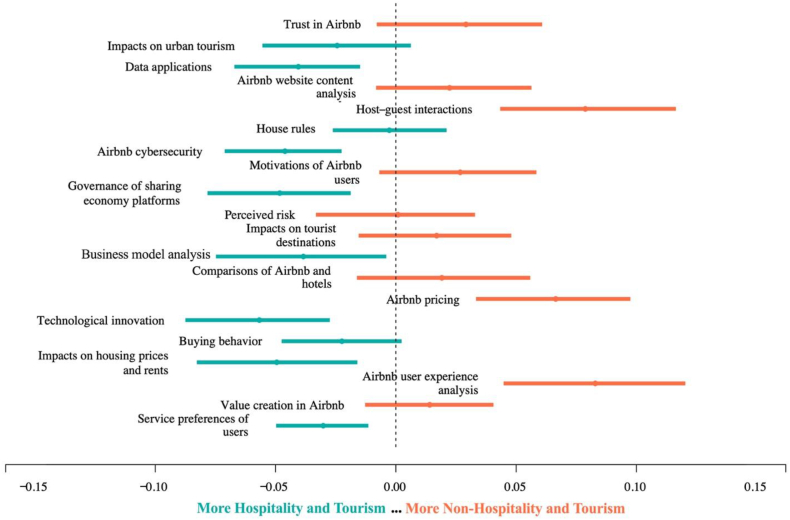
Change in topic prevalence based on the journal field. Note: The marginal effects depicted in this figure pertain to the change of expected proportions of topic prevalence based on the distinction between two journal fields: hospitality and tourism journals, and non-hospitality and tourism journals. Source: Created by the authors.

Fig. 6 depicts the marginal effect of the journal field on the distribution of 20 topics, where the mean values of the estimated differences are represented by dots, and the 95% confidence intervals of the differences are shown as bars. The placement of the topics on the plot reflects their likelihood of appearing in either hospitality and tourism journals or non-hospitality and tourism journals. Specifically, topics located towards the left of the plot are more likely to appear in non-hospitality and tourism journals, while those on the right are more likely to appear in hospitality and tourism journals. The findings reveal that certain topics, such as ‘trust in Airbnb,’ ‘perceived risk,’ ‘host-guest interactions,’ ‘value creation,’ and ‘Airbnb user experience analysis’ are more frequently discussed in hospitality and tourism journals. These topics are primarily related to research on Airbnb user behavior. On the other hand, topics that appear more frequently in non-hospitality and tourism journals cover multiple research areas, including business analysis (e.g., ‘technological innovation’ and ‘business model analysis’), P2P platform management (e.g., ‘house rules,’ ‘governance of sharing economy platforms,’ and ‘Airbnb cybersecurity’), and the impact of Airbnb on various domains (e.g., ‘impacts on urban tourism’ and ‘impacts on housing prices and rents’).

### 3.8 Topic correlation analysis

**Fig. 7:**
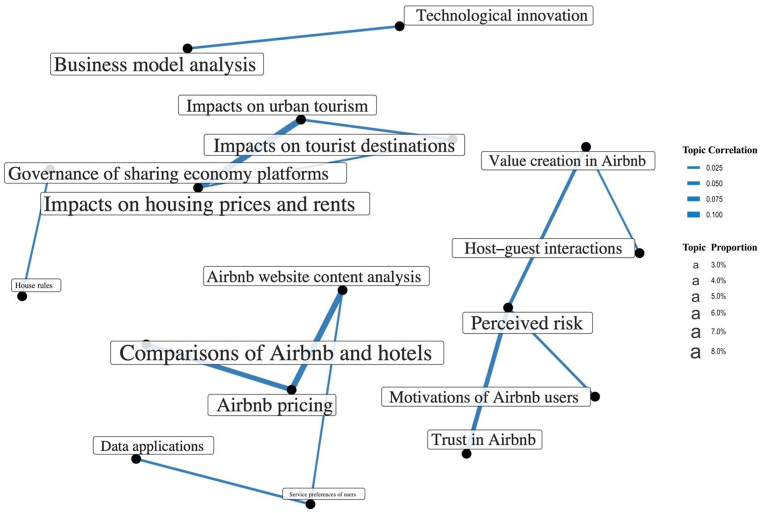
Topic correlation map. Note: The thickness of the connecting lines represents the strength or closeness of the connections between the topics, while the size of the text corresponds to the percentage representation of each topic. Source: Created by the authors.

Fig. 7 presents a map of the intercorrelations among the extracted topics. Three topics, specifically ‘Airbnb cybersecurity,’ ‘buying behavior,’ and ‘Airbnb user experience analysis,’ are not included in the topic correlation map due to their weak correlation with other topics in the analysis. The thickness of the connecting lines represents the strength of the correlation between two topics, while the size of the label font indicates the relative proportion of the topic. The topics are grouped into five clusters, each with distinct characteristics. Two clusters consist of only two topics: ‘technological innovation’ and ‘business model analysis,’ and ‘governance of sharing economy platforms’ and ‘house rules.’ These clusters explore the dynamics of the sharing economy and the regulatory mechanisms that govern it, highlighting the significance of sound business practices and innovation in the sharing economy. Three topics in the cluster focus on Airbnb's impact on different domains, including ‘impacts on urban tourism,’ ‘impacts on tourist destinations,’ and ‘impacts on housing prices and rents.’ These topics delve into the social and economic implications of Airbnb's operations and underscore the importance of understanding the impacts of short-term rentals on local communities. The cluster with five topics centers on ‘Airbnb pricing,’ which highly correlates with ‘comparisons of Airbnb and hotels’ and ‘Airbnb website content analysis.’ These topics examine pricing strategies in the sharing economy, compare Airbnb's pricing with traditional hotel pricing, and investigate the influence of Airbnb's website content on consumer behavior. ‘Value creation in Airbnb’ and ‘perceived risk’ are the representative topics of the last cluster, which focuses on research on Airbnb users. These topics investigate the factors that motivate consumers to use Airbnb and the perceived risks associated with participating in the sharing economy.

### 3.9 Topic trend analysis

**Fig. 8:**
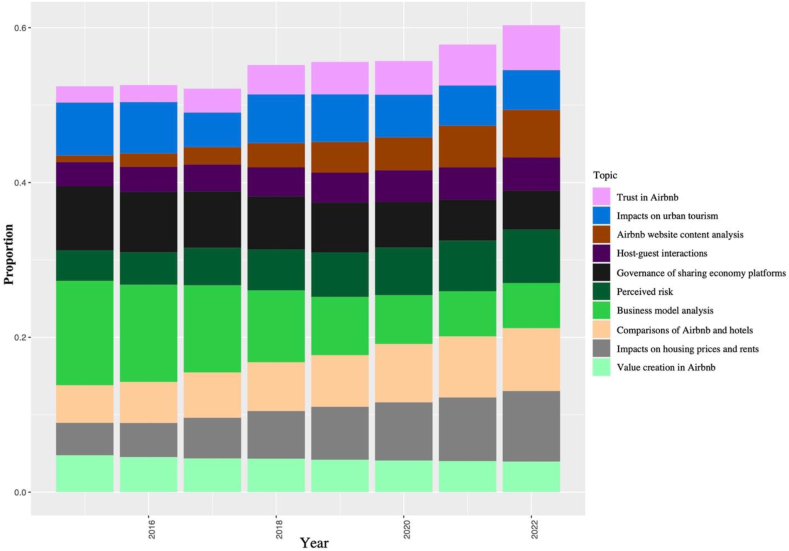
Annual trends of the selected topics. Note: The annual trends depicted in this figure illustrate the temporal evolution of the selected topics between 2015 and 2022. Source: Created by the authors.

Fig. 8 displaces the changing patterns of representative topics from different clusters, as presented in Fig. 7, from the year 2015–2022. The proportional values were estimated using the “STM” package's *estimateEffect* function, which is widely used in topic modeling analysis. The results demonstrate that half of the ten representative topics showed an upward trend, indicating a sustained interest in these topics over the years. These topics include ‘trust in Airbnb,’ ‘Airbnb website content analysis,’ ‘perceived risk,’ ‘comparisons of Airbnb and hotels,’ and ‘impacts on housing prices and rents’. In contrast, the remaining five topics experienced a decline in research interest over the same period. These topics include ‘impacts on urban tourism,’ ‘host-guest interactions,’ ‘governance of sharing economy platforms,’ ‘business model analysis,’ and ‘value creation in Airbnb.’ The changing trends in research interest reflect the evolving landscape of the sharing economy and the growing impact of Airbnb on various sectors. The changing trend of each topic can be further explored to gain a more in-depth understanding of the issues at hand. The evolving pattern of each topic extracted in this study is illustrated in Fig. S2, which can supplement the content in Fig. 8.

## 4 Discussion

Through the analysis of the 20 topics extracted in this study, it is found that the major academic concerns regarding Airbnb are related to its operational practices and impacts on various fields. Concerning operational practices, the most relevant research topics include ‘trust in Airbnb,’ ‘house rules,’ ‘Airbnb pricing,’ and ‘value creation in Airbnb.’ In-depth investigations on these subjects provide Airbnb with valuable insights for formulating effective property management policies, while also equipping hosts with practical strategies to optimize their P2P rental businesses. In the realm of Airbnb users, six prominent research topics have emerged: ‘host-guest interactions,’ ‘perceived risk,’ ‘service preferences of users,’ ‘buying behavior,’ ‘motivations of Airbnb users,’ and ‘Airbnb user experience analysis.’ Among these topics, studies centered around user experience are the most prevalent, delving into diverse facets of Airbnb users' lodging experiences. As Airbnb's accommodation services evolve, users have increasingly diverse expectations, prompting research that explores different aspects of user needs. For instance, there are studies comparing the needs of different cross-cultural Airbnb users, expectations of users selecting different Airbnb accommodation products, and analyses of changing trends in user preferences.

As for Airbnb's impacts, the related research topics include ‘impacts on urban tourism,’ ‘impacts on housing prices and rents,’ ‘impacts on tourist destinations,’ and ‘comparisons of Airbnb and hotels.’ Among the studies related to tourism, researchers have reported the positive and negative impacts of Airbnb. Previous studies have found that for well-known tourist destinations, the boosting effect of Airbnb on tourism has not been well received by local residents, as over-tourism brought by Airbnb has become an important factor of dissatisfaction for residents of these destinations. In contrast, for some developing countries, Airbnb is widely recognized as a boost to tourism, and it is supported by government agencies in certain countries [73], as it not only promotes the development of the tourism economy but also alleviates the employment pressure of the local population to a certain extent. Another area of research that has received a lot of attention from researchers is the impact of Airbnb on the traditional hotel industry. By the very nature of the industry, the two types of accommodations are in direct competition with each other, but findings from previous studies are inconsistent. Researchers have found that Airbnb's impact on the traditional hotel industry is influenced by a very large number of factors, such as geography, hotel category, and location of the hotel, making it difficult to make direct inferences that Airbnb has a direct impact on the traditional hotel industry. The remaining research topics include ‘data applications,’ ‘governance of sharing economy platforms,’ ‘Airbnb cybersecurity,’ ‘technological innovation,’ and ‘business model analysis.’

The utilization of surveys as a research method has persisted as a popular approach among scholars investigating the Airbnb phenomenon [53,55,64,66]. Meanwhile, the application of regression analysis based on Airbnb listing data has also garnered significant attention from researchers [58,59,62,69]. In order to obtain such data, scholars often depend on AirDNA [59,62,77], a reputable data analytics enterprise that charges for its services. On the other hand, InsideAirbnb (www.insideairbnb.com) is a freely available resource that many researchers employ to gather research data [12,45,58]. Nevertheless, the reliability of the data from InsideAirbnb is a matter of concern [74]. Alsudais [74] revealed two significant issues with the dataset's quality, which are likely due to systemic errors in the data collection process. In addition, the reproducibility of research outcomes may be compromised when comparing different iterations of the dataset. Therefore, it is suggested that researchers must exercise caution when using open datasets and thoroughly assess the data's validity before drawing any conclusions [74].

Research on Airbnb has been published in both hospitality and tourism journals as well as non-hospitality and tourism journals, and we have identified some differences in the emphasis of topics in these two types of journals. Hospitality and tourism journals tend to prioritize topics such as “host-guest interactions,’ ‘Airbnb user experience analysis,’ and ‘Airbnb pricing’. These topics are of significant interest to researchers in the hospitality and tourism field as they directly relate to the industry. Conversely, non-hospitality and tourism journals demonstrate a broader scope of interest regarding Airbnb research. Examples of such topics include ‘Airbnb cybersecurity,’ ‘governance of sharing economy platforms,’ and ‘impacts on housing prices and rents.’ These areas of investigation reflect the interdisciplinary nature of the sharing economy phenomenon and its implications for various fields such as technology, law, and urban planning. While there is some overlap in the topics covered by these two types of journals, the differences reflect the disciplinary focus of the respective journals.

The findings of this study suggest that there has been a shift in focus in Airbnb-related research over time. The topics that have shown significant growth in prevalence, such as ‘trust in Airbnb,’ ‘Airbnb user experience analysis’ and ‘Airbnb pricing,’ indicate that there is increasing attention on issues related to user experience and pricing strategies on Airbnb. Among the studies related to Airbnb user experience, we found that in addition to the increasing hotness of this research topic, there are significant changes in the methods of studying user experience. The main factor leading to the change in research methods is the continuous development of big data technology, which makes it possible to analyze a large scale of data efficiently. In Airbnb user experience-related research, many scholars applied text mining techniques to analyze user reviews posted on the platform to obtain important information, as these reviews often contain detailed descriptions of users' lodging experiences. To draw insight into these unstructured big data, many different text mining techniques have been applied, such as Latent Dirichlet Allocation (LDA) [45], Latent Semantic Analysis (LSA) [46], and STM [75]. Meanwhile, the significant declines in topics such as ‘business model analysis,’ ‘governance of sharing economy platforms,’ and ‘technological innovation’ suggest that research attention on the sharing economy and related technologies may be waning. The stability of the prevalence of ‘value creation in Airbnb’ may suggest that this topic is well-established and is of enduring interest to researchers. The findings also suggest that issues related to the impact of Airbnb on local residential areas and tourist destinations continue to be significant areas of interest for researchers. This is consistent with the ongoing debates around the effects of Airbnb on local housing markets, communities, and tourism [68,76]. The trends in the prevalence of different Airbnb-related research topics reflect the dynamic nature of the sharing economy and the evolving concerns and interests of researchers in this field.

This study contributes to the existing literature on Airbnb research by offering a comprehensive review of recent studies in the field. This study quantifies the notable increase in the publication of Airbnb articles over the past seven years, indicating rapid growth in research interest in the platform. From the perspective of methodology, this study extends previous Airbnb literature reviews by applying STM in the review process, effectively addressing the common limitations associated with traditional qualitative reviews. For instance, this study employed larger samples with a wider scope, thereby reducing potential literature selection biases. By expanding the scope, this study encompasses a diverse set of Airbnb-related topics, offering insights into various dimensions of the platform. Specifically, this study extracts 20 Airbnb-related research topics from these documents, which contributes to a more nuanced understanding of the research landscape surrounding Airbnb. In addition, the methodology employed in this study enables the acquisition of comprehensive insights from a substantial corpus of research papers in a prompt and efficient manner. This efficiency is particularly valuable in a rapidly evolving field like Airbnb, where staying up-to-date with the latest research is crucial. Therefore, the detailed steps for applying STM presented in this study serve as a useful reference for future literature reviews on Airbnb. By utilizing the advantages of STM, this study further investigates how these research topics vary across hospitality and tourism journals as well as non-hospitality and tourism journals by incorporating document metadata information. Furthermore, this study contributes to the identification of underlying trends that change Airbnb research priorities over time. For instance, the increasing prevalence of research topics such as ‘trust in Airbnb’ and ‘impacts on housing prices and rents’ indicates a growing interest in the social and environmental effects of Airbnb. This trend may prompt more research on the company's social responsibility and sustainability practices. Conversely, the decreasing prevalence of topics such as ‘business model analysis’ and ‘technological innovation’ suggests that these areas have been extensively studied, urging future research to explore new avenues of inquiry.

This study has implications not only for the academic literature on Airbnb research but also for practitioners in the Airbnb business and policymakers responsible for regulating the platform. The findings of this study can provide insights and guidance to those involved in the Airbnb business regarding emerging trends in research and areas where future research could be focused. By keeping up with the latest research, practitioners can better understand the evolving expectations and demands of their customers and make informed decisions on how to optimize their business operations. Furthermore, policymakers and regulatory bodies can benefit from the insights provided by this study to better understand the potential impact of sharing economy platforms like Airbnb on their industries. With the increasing popularity of Airbnb, concerns have been raised about its impact on rental housing markets, local communities, and traditional hotels. This study can inform policymakers about the potential risks and benefits associated with Airbnb and provide recommendations for adapting regulations to ensure the safety and security of consumers and the maintenance of fair competition in the marketplace. Policymakers can also use this study to evaluate the effectiveness of existing regulations and identify areas where changes may be necessary to ensure that the sharing economy operates in a socially responsible and sustainable manner.

## 5 Conclusion

Researchers have made considerable efforts to explore Airbnb from various perspectives. However, the sheer volume and increasing diversity of Airbnb studies present challenges in identifying major research topics and emerging trends through traditional qualitative analysis methods alone. This study fills the gap by providing a comprehensive overview of existing Airbnb research, leveraging the methodological advantages offered by text-mining techniques. By employing these techniques, this study identified two prominent academic concerns regarding Airbnb, namely operational practices and impacts on various fields. Concerning operational practices, the focus encompasses multiple facets of running a peer-to-peer accommodation business. This includes aspects such as pricing strategies, formulating comprehensive accommodation rules to ensure safety and security, as well as developing effective strategies for enhancing the overall user experience. Moreover, the impacts of Airbnb on urban tourism, housing prices and rents, tourist destinations, and the traditional hotel industry have been subjects of extensive investigation. This study sheds light on these impacts, presenting a nuanced understanding of the complex relationships between Airbnb and these sectors. It is noteworthy that the research focus has evolved over time, with a shift from macro-level analyses to micro-level aspects. Notably, there has been an increasing emphasis on understanding user experiences and pricing strategies, reflecting the growing significance of these factors in shaping the Airbnb ecosystem. Conversely, topics such as business model analysis and technological innovation have seen a decline in research attention. Furthermore, this study reveals an interesting disparity between research articles published in non-hospitality and tourism journals compared to those in hospitality and tourism journals. Articles in non-hospitality and tourism journals cover a more diverse range of topics, reflecting the interdisciplinary nature of Airbnb research. This diversity suggests that scholars from various disciplines recognize the relevance and impact of Airbnb beyond the boundaries of traditional hospitality and tourism research.

While this study holds considerable implications, it is important to acknowledge its limitations when interpreting the results. The conclusions drawn are based on a specific selection of journals, and only abstracts were analyzed, potentially leading to an incomplete representation of the research papers’ content. Despite the wide coverage of the Scopus database, there is a possibility of overlooking relevant studies published in non-indexed journals. The exclusion of studies published in non-indexed journals raises the possibility of missing valuable insights that could have enriched the analysis. Moreover, certain topics identified in the analysis may encompass sub-topics that were not fully captured by the designated names of those topics, limiting the comprehensive understanding of the themes discussed in the full texts of the articles.

The findings of this study provide valuable insights and suggest several directions for future research. Previous studies on Airbnb underscore the significance of understanding regional characteristics within the platform [14,18,45]. To advance this understanding, future research should conduct comparative studies that classify regions based on their distinct characteristics. For instance, comparisons between cities in developed and developing countries or among different developed countries would shed light on variations in Airbnb dynamics. As the P2P accommodation industry grows more competitive, prioritizing customer experience is paramount for the sustainable development of the business [63]. To comprehend and cater to the diverse needs of various market segments on Airbnb, researchers should adopt an interdisciplinary approach in exploring user preferences rather than relying solely on generalizations [73]. Incorporating perspectives from fields like anthropology, sociology, and psychology can yield a more comprehensive understanding of users, unveiling nuanced insights. This knowledge can serve as a valuable reference for practitioners to create personalized and engaging customer experiences. Furthermore, with the advent of technologies like machine learning and artificial intelligence, researchers can investigate the potential for Airbnb to enhance user experiences through design and technological innovations. Moreover, as the P2P accommodation market continues to mature, an increasing number of professional hosts are joining the platform [77]. However, there is currently limited understanding of user perceptions of professional management in Airbnb accommodations. The question of whether a professionalized Airbnb experience can lead to further business growth is worthy of exploration, as the distinction between a standardized Airbnb and traditional hotels will further decrease. With sustainability becoming increasingly important in the sharing economy [78], researchers can investigate the role of Airbnb in promoting sustainable and eco-friendly tourism, as well as its impact on local communities and the environment. As Covid-19 restrictions ease and tourism destinations reopen, it is urgent to address the challenges posed by Airbnb. In this context, conducting studies on effective tourism destination management becomes crucial for policymakers, industry practitioners, and researchers.

## Author contribution statement

All authors listed have significantly contributed to the development and the writing of this article.

## Funding statement

This research is supported by the Start-up funds for scientific research of high-level talents from Ningbo University of Finance and Economics (Grant No. 100064/1320229064).

## Data availability statement

Data will be made available on request.

## Declaration of competing interest

The authors declare that they have no known competing financial interests or personal relationships that could have appeared to influence the work reported in this paper.
