# Supplementary material for: The evolution of Airbnb research: A systematic literature review using structural topic modeling
Source: Heliyon. 2023 Jun 8;9(6):e17090. doi: 10.1016/j.heliyon.2023.e17090 (PMC10361235; doi:10.1016/j.heliyon.2023.e17090)
Supplement: Multimedia component 1 [file mmc1.docx]

**Supplemental Figure legends**

**Fig. S1.** Topic quality.

**
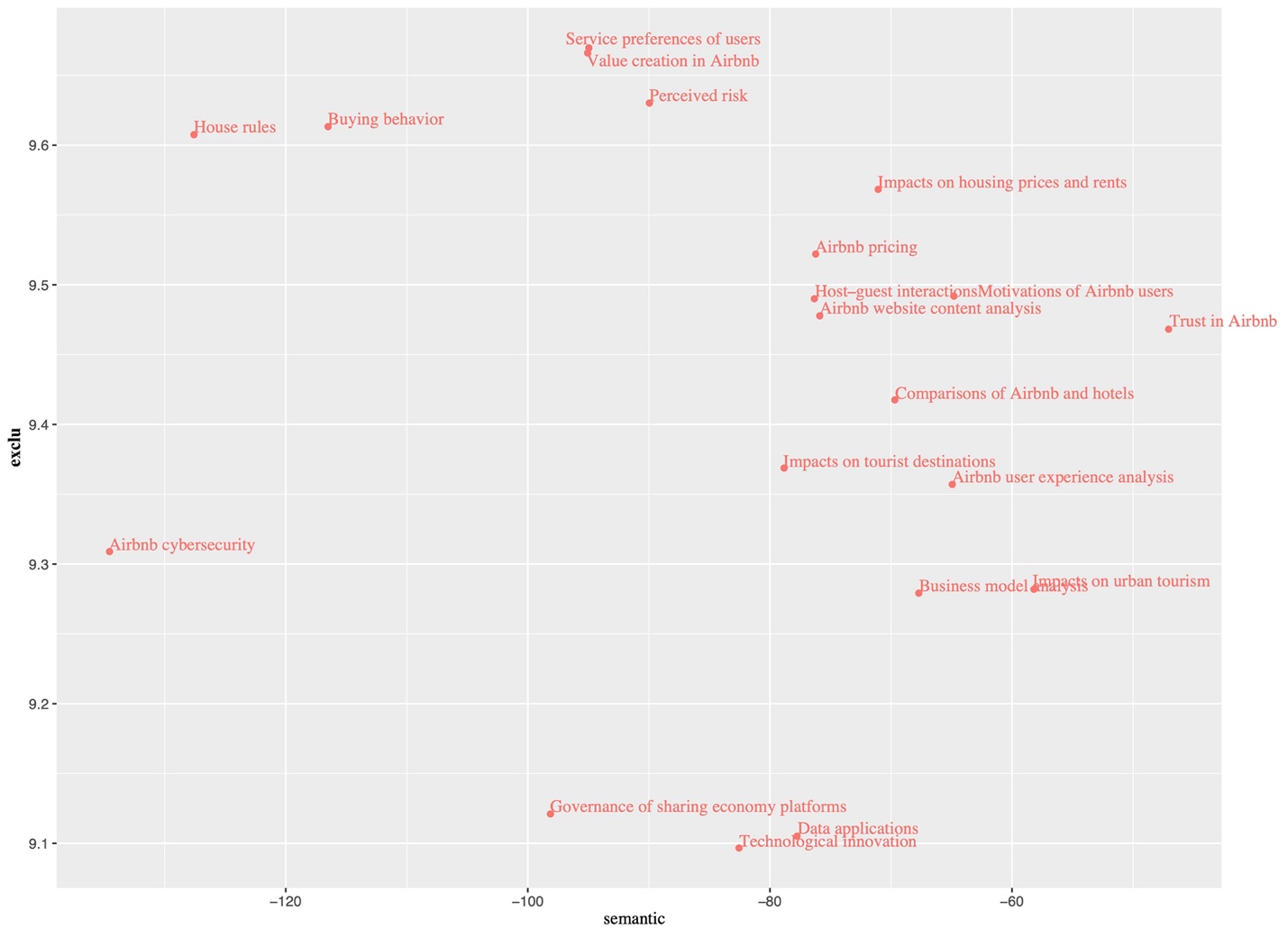
**Note: The closer a topic is to the upper right corner, the better its performance in terms of exclusivity and semantic coherence. This means that topics that are located in this area are more distinct from other topics and have a stronger internal consistency in terms of the words and concepts used.

Source: Created by the authors.

**Figure S2.** Topic trend.


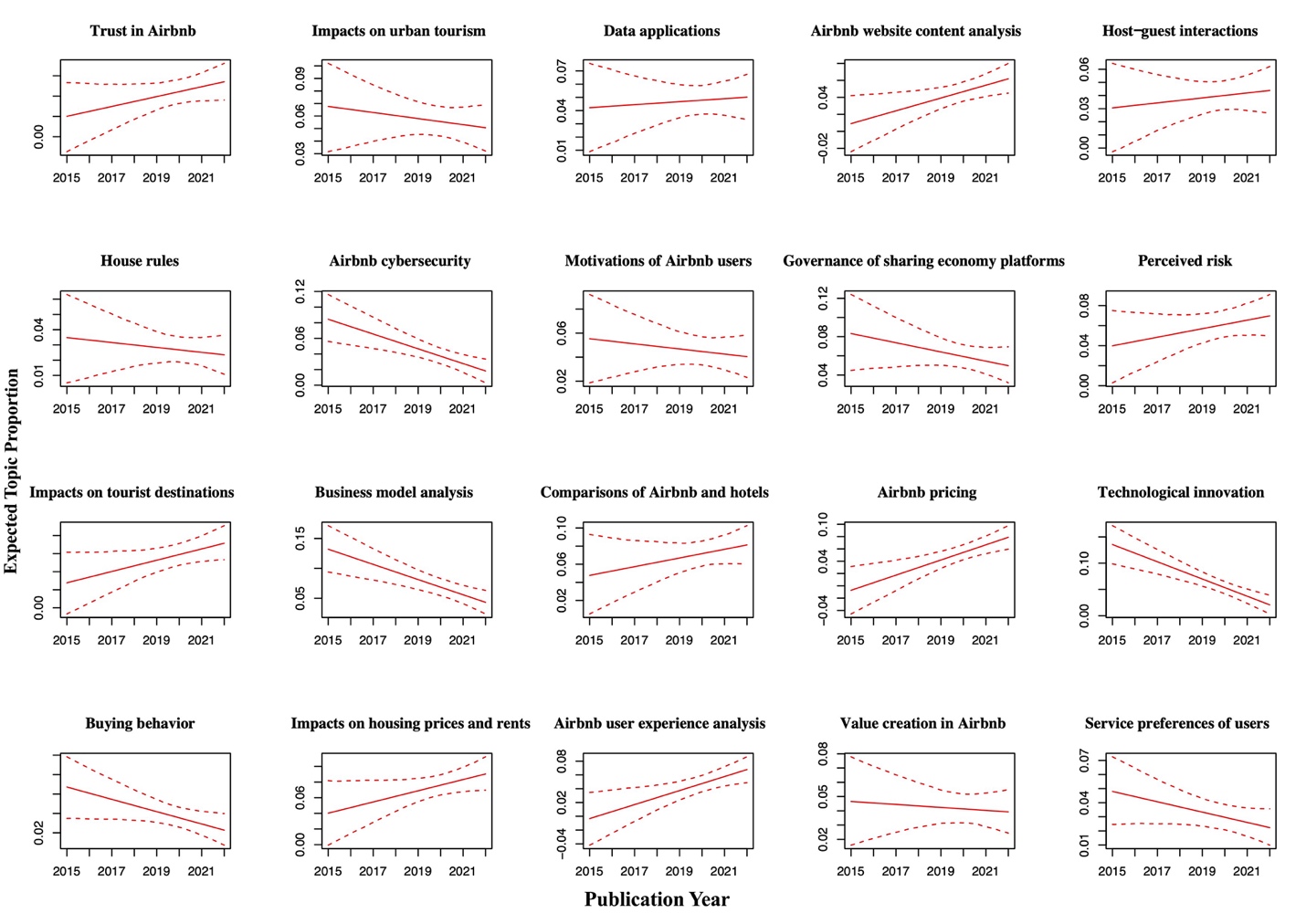


Note: If the solid line is going upwards, it indicates that the level of attention to this topic in research is continuously increasing over time. Conversely, if the line is going downwards, it means that the topic is losing interest or relevance in the field. These trends can provide valuable insights into the direction and evolution of research in a particular area.

Source: Created by the authors.
